# Comparison of Fluoride Levels in Tap and Bottled Water and Reported Use of Fluoride Supplementation in a United States–Mexico Border Community

**DOI:** 10.3389/fpubh.2017.00087

**Published:** 2017-04-27

**Authors:** Kerton R. Victory, Nolan L. Cabrera, Daniela Larson, Kelly A. Reynolds, Joyce Latura, Cynthia A. Thomson, Paloma I. Beamer

**Affiliations:** ^1^Mel and Enid Zuckerman College of Public Health, The University of Arizona, Tucson, AZ, USA; ^2^College of Education, The University of Arizona, Tucson, AZ, USA; ^3^Mariposa Community Health Center, Nogales, AZ, USA

**Keywords:** low-income families, fluoride, bottled water, tap water, dental caries, United States–Mexico border, Latino, health disparities

## Abstract

**Background:**

Compared to the general United States (U.S.) population, Arizona counties along the U.S.–Mexico border have a higher prevalence of dental caries, which can be reduced with adequate fluoride exposure. Because of concern regarding local tap water quality, fluoride-free bottled water consumption is common in this region, raising concern that families are not receiving adequate fluoride to promote dental health.

**Objective:**

To evaluate the levels of fluoride in tap and bottled water as well as the use of fluoride supplements in an Arizona border community.

**Methods:**

Low-income Latino households (*n* = 90) who report use of bottled water as their primary source of water intake were recruited. Participants completed a questionnaire about their and their children’s dental histories and use of fluoride supplements. Water samples (bottled and tap) were collected from a subset of households (*n* = 30) for analysis of fluoride.

**Results:**

Fluoride detection levels were significantly greater (*p* = 0.02, Fisher’s exact test) in tap water (average = 0.49 mg/dL) than in bottled water, yet, the majority (22/30) were below the range for optimal dental health (0.7–1.2 mg/L). Concentration of fluoride in the majority (29/30) of bottled water samples was below the quantitative detection limit of 0.4 mg/L. Children were significantly less likely to have dental caries if they received fluoride varnishing treatments (*p* = 0.01, Fisher’s exact test), lived in households that reported using fluoridated mouthwash (*p* < 0.001, Fisher’s exact test), their parents received fluoride education (*p* = 0.01, Fisher’s exact test), and their parents reported visiting a dentist yearly (*p* < 0.001, Fisher’s exact test). Furthermore, none of the participants reported receiving recommendations from health-care providers about fluoride supplementation or variance in content by the type of water consumed.

**Conclusion:**

Although fluoride was significantly more likely to be detected in tap than bottled water, neither water source in this border community is likely to provide enough fluoride for optimal dental health. Low-income children in this region may benefit from regular access to fluoride varnishing treatments and/or use of fluoridated mouthwash, interventions that could be tested in future well-designed trials.

## Introduction

Dental caries remain a public health problem for many developing countries and for underserved populations in developed countries (1, 2). Exposure to fluoride can reduce dental caries and enhance remineralization of early carious lesions (3). While fluoride can be administered *via* toothpaste, mouthwash, nutritional supplements, and topical treatments such as varnish, the primary approach has been to fluoridate municipal water supplies. The American Dental Association (ADA) recommends that fluoride levels in drinking water should be between 0.7 and 1.2 mg/L for optimal dental health. Because too much fluoride is associated with mottled teeth and potentially bone disease, the U.S. Environmental Protection Agency (USEPA) mandates that the primary maximum contaminant level (MCLG) for fluoride in drinking water is 4.0 mg/L, with a secondary maximum contaminant level of 2.0 mg/L in areas that have high levels of naturally occurring fluoride (4). Fluoridation of municipal tap water began over six decades ago in the United States (U.S.) and approximately 60% of the population has access to optimally fluoridated water (i.e., 0.7–1.2 mg/L) (5).

Several epidemiological studies and systemic reviews have demonstrated that consumption of fluoridated water is associated with improved dental health (6–8). Over the past 60 years, the ADA has continued to endorse fluoridation of municipal water supplies and have reported significant reductions in caries among children aged 5–17 (3). Additionally, a study conducted by the Centers for Disease Control and Prevention (CDC) in the U.S. from 1967 to 1999 showed that 12-year-old children who lived in areas with fluoridated water had fewer decayed, missing (because of carries), or filled permanent teeth than those who lived in areas without fluoridated water (9, 10).

In several countries where fluoride is not added to municipal drinking water supplies, a substantial decline in the prevalence of caries has still been reported, with reductions in lifetime caries exceeding 75% (11). The main reason for this decline can be attributed to the introduction of fluoridated toothpaste and to increased public health programs that provide fluoride varnishing treatments to children (1, 12). However, when compared to countries where fluoride is added to municipal drinking water supplies, the prevalence of caries is still about 20–30% higher (13).

In some areas, fluoride is not added to drinking water because levels of naturally occurring fluoride might be markedly higher (>1.5 mg/L) than the ADA recommended range for optimally fluoridated water (4, 14, 15). Furthermore, one study demonstrated that the risk for dental fluorosis significantly increases with levels of fluoride above 1.5 mg/L in drinking water (16). In regions where dental fluorosis is endemic, consumers are advised to drink alternative sources of water including bottled water (16). In the U.S., most people have access to optimal fluoridated drinking water; however, many residents may be receiving lower levels of fluoride in their drinking water for optimal dental health if they primarily consume bottled water (17).

In particular, concerns have been raised over the increased consumption of bottled water in the U.S. and its impact on oral health (18, 19). Although the maximum contaminant level for fluoride in public drinking water is 4.0 mg/L. Multiple studies have demonstrated that fluoride is not usually detectable in bottled water, and when it is, the concentrations are not within the range recommended by the ADA for optimal dental health (5, 17).While a few companies in the U.S. offer fluoridated bottled water as an alternative, many consumers may not be aware of the benefits associated with fluoridated bottled water (20–22). Similarly, many bottled water drinkers may not be aware of the recommended use of additional fluoride supplementation if they primarily consume bottled water. While for some, consumption of bottled water may be a convenience or status symbol (5), many lower socioeconomic status (SES) populations rely on bottled water as their primary drinking water source because of concern regarding the safety of their tap water (23–25). In particular, Latinos are more likely than non-Latinos to give their children bottled water because they fear that drinking tap water might result in illness (26, 27).

Despite the overall reduction of dental caries in the US population, marginalized and low-income populations continue to experience a disproportionate burden (28). While some of this may be attributable to diet or other risk factors, several studies have reported that water fluoridation is effective in reducing dental caries in populations of lower SES (23, 29). For example, in a study of 6,638 children that were of 12 years of age, researchers concluded that fluoride supplementation resulted in a 37% reduction in tooth decay in children from families with socio-economic dental health inequalities (29). In the most recent National Health and Nutrition Examination Survey, 23 and 56% of US children aged 2–5 and 6–8 years, respectively, have experienced dental caries (30). Hispanic and Black children have significantly higher rates. Increased efforts are needed to enhance fluoride supplementation and education to these populations.

In the state of Arizona, the rate of dental caries among children is higher than the U.S. average with 37 and 60% of Arizona children aged 2–4 and 6–8 years, respectively, having experienced dental caries (31, 32). Of particular concern is that all four Arizona counties that border Mexico have rates that exceed the state average, with Santa Cruz county reporting the highest rate where 68% of 6- to 8-year-old children have dental caries (31). The Binational Health Commission in their Healthy Border 2020 Plan has identified maternal and child health as one of the main public health priorities for this unique geographic region (33). More research is needed to better understand if dental caries are higher across this region and what risk factors are associated with this potential border health disparity so that better interventions can be designed.

While there are many potential risk factors in Santa Cruz County that may explain increased prevalence of dental caries in children, one of the contributing factors may be insufficient fluoride consumption for prevention of tooth decay. The primary city in Santa Cruz County is Nogales. Fluoride is not added to tap water in Nogales because it comes from groundwater with naturally occurring fluoride in the range of 0.22–2.90 mg/L (34). While this overlaps with the range recommended by ADA of 0.7–1.2 mg/L, it is not clear if Nogales residents are receiving sufficient fluoride in their tap water. In our previous work, we documented that 85% of low-income families in Nogales exclusively drink bottled water and half cook with bottled water (25). Additionally, in another study of the U.S.–Mexico border, researchers demonstrated that environmental issues, including frequent groundwater contamination with microbial and chemical agents in addition to water shortages, have resulted in increased bottled water consumption in this region (35). Thus, it is not known if residents are receiving adequate levels of fluoride in either tap or bottled water for optimal dental health or if they are using fluoride supplementation.

The primary purpose of this study was to assess levels of fluoride in tap water and bottled water in Nogales, AZ, USA, and evaluate the use of fluoride supplementation by low-income Latino families that primarily drink bottled water. While others have compared the levels fluoride in tap and bottled water (5, 26), this has not been assessed in a region that does not add fluoride to their tap water. Secondary objectives of this study were to determine what factors such as fluoride education or advice from healthcare professionals might be associated with decreased caries in children and adults in this underserved region. This exploratory study provides new insights into understanding fluoride exposure among low-income Latino families in the U.S.–Mexico border region, so that better interventions can be developed to reduce dental health disparities in this population.

## Materials and Methods

### Study Population and Recruitment

A total of 90 low-income Latino households from Nogales, AZ, USA, participated in this study. Additionally, water samples were collected from 30 of these households for analysis of fluoride. Participants were recruited during regular business hours from Mariposa Community Health Center (MCHC) waiting rooms, a chain discount store, and participant referrals. MCHC is a federally qualified health center that is a major provider of medical, dental, and preventive care in Santa Cruz County and other public health services common to county health departments. To meet the inclusion criteria, families had to have an annual household income less than $30,000, be connected to a public water utility, primarily drink bottled water, and have at least one child living in the home. Individuals whose households were supplied mainly by water from private wells or who primarily drank their tap water were excluded. Families who primarily drink bottled water were selected because they are potentially most at risk for less fluoride in their water and increased dental caries, may be more likely to use fluoride supplementation and may be the most important group to target for an intervention.

### Water Sample Collection and Analysis

One liter of tap water and 1 L of bottled water were collected from each of the 30 homes in Nogales, AZ, USA for measurement of fluoride. Prior to water collection, the area around the faucet was cleared of any objects (e.g., dishes, containers) which could obstruct the flow of water. The cold water faucet was turned on and run for approximately 1–2 min. Water was collected in sterile, 1-L sample bottles and immediately capped. Samples were stored on ice in a cooler (~4°C) and transported to Test America, Inc., an Arizona state certified environmental testing laboratory. Fluoride levels in each sample were assessed using USEPA Method 300.0 with ion chromatography and a detection limit of 0.4 mg/L (36).

Chain of custody forms were completed and submitted to the lab. As required by USEPA Method 300.0, a trip blank and temperature indicator were taken to the sampling location each time to guarantee that primary samples were not contaminated during transport. Quality control samples (QCS) were immediately processed after calibration of every 10 samples throughout the entire analytical batch. The measured results for the QCS were compared to the true values to determine percent recoveries. If QCS results were not within ±10% of the true value, the analysis was terminated, the problem corrected, and the analysis repeated.

### Questionnaire

A questionnaire was developed from several drinking water studies (1, 26, 37) and administered orally in Spanish (75%) or English (25%), according to each participant’s language preference. Participants completed the questionnaire in approximately 20–30 min. Residents were asked about their use of topical and systemic fluoride supplements, dental histories, their children’s dental histories, and whether they had been provided with drinking water recommendations by their dental or healthcare providers or informed about the importance of fluoride and/or fluoride supplementation.

### Data Analysis

All questionnaire responses and fluoride water concentrations were hand coded into STATA^®^ (version 12.1, College Station, TX, USA), which was used for all statistical analyses. Fisher’s exact test was used to determine whether there was a significant difference in the detection levels of fluoride in tap and bottled water. Fisher’s exact test was also used to determine whether significant associations existed between caries outcomes in children or parents and prior knowledge about the importance of fluoride along with several other participant characteristics, including language in which the survey was administered, immigration status of respondent (immigrant and non-immigrant), number of years respondent had been living in the U.S., annual income level of household, education level of respondent, prior dental visits reported by respondent, or whether their child or children had received fluoride varnishing treatments.

## Results

Fluoride detection levels in tap water were significantly higher (*p* = 0.02, Fisher’s exact test) than those in bottled water. Fluoride was detected in 50% (15/30) of the tap water samples but only in one bottled water sample (Table 1). Tap water collected from homes supplied by water purveyor A had detectable levels of fluoride, whereas samples collected from homes supplied by water purveyors B and C were below the limit of detection of 0.4 mg/L (Table 1). In tap water samples where fluoride was detected, only 53% (8/15) contained fluoride levels within the recommended ADA range for optimal dental health indicating that 73% (22/30) of the tap water samples collected in Nogales do not meet the ADA recommendations (Table 1). The range of fluoride concentration was 0.58–1.10 mg/L and all samples were below the U.S. EPA maximum contaminant level (Table 1). Thus, although tap water had higher amounts of fluoride than bottled water, in the Nogales region, drinking tap water alone is not likely to provide enough fluoride to promote optimal dental health. Interventions are needed to increase fluoride intake regardless of the families’ drinking water source.

**Table 1:** **Summary of detection levels of fluoride in bottled and tap water from 30 households in Nogales, AZ, USA**.

Water type	Detection frequency (%)	Below ADA range (%)	Mean	SD	Min	P25	P50	P75	Max
Bottled water	3 (1/30)	97 (29/30)	_	_	ND	ND	ND	ND	0.72
Tap water	50 (15/30)	73 (22/30)	0.49	0.17	ND	0.40	0.40	0.59	1.10
Tap water purveyor A	79 (15/19)	47 (7/15)	0.58	0.21	ND	0.40	0.65	0.70	1.10
Tap water purveyor B	0 (0/4)	100 (4/4)	ND	ND	ND	ND	ND	ND	ND
Tap water purveyor C	0 (0/7)	100 (7/7)	ND	ND	ND	ND	ND	ND	ND

*For tap water, samples with ND were replaced with the LOD/√2 for calculation of mean and SD*.

*ADA, American Dental Association; ND, non-detect (below detection limit)*.

The majority of the respondents answered the questionnaire in Spanish (Table 2). All of our participants were Latino, with about half being immigrants. However, the majority of participants have lived in the U.S. for >10 years. About half of the families reported an annual income of <$15,000. The majority of parents did not attend college with approximately 30% completing high school or its equivalent. Most of the parents (86%) reported having dental caries, and 58% of the households reported having at least one child with dental caries. Only half of the children received fluoride varnish treatments, and less than half of the households reported using mouthwash with fluoride. Interestingly, 34% of the parents reported having never visited a dentist, indicating that access to dental health professionals may be a concern for this population. Only a third of parents reported having received some education on the importance of fluoride for dental health, yet none of the 90 participants received any recommendations from any healthcare or dental providers about the types of water they drank or fluoride supplementation. Thus, it is likely that many of these low-income Latino families may not be aware of the potential increased risks of dental caries related to the lack of fluoride in their drinking water.

**Table 2:** **Association between any children in the household having had dental caries with the household’s demographic and dental hygiene characteristics**.

			Dental caries				
	Overall		Yes		No		
Characteristic	*n*	%	*n*	%	*n*	%	*p*-Value
**Demographics**							
Survey language							0.42
Spanish	72	80	38	83	27	73	
English	18	20	8	17	10	27	
Immigrant parents							0.38
Yes	44	47	22	48	22	59	
No	46	53	24	52	15	41	
Years parents have lived in the United States							0.78
0–10	22	24	11	24	8	22	
11–20	35	39	19	41	13	35	
>20	33	37	16	35	16	43	
Household income							0.66
<$15,000	48	53	26	56	19	51	
$15,000–$30,000	42	47	20	44	18	49	
Parents’ highest level of education							1.0
8th grade	43	48	22	48	17	46	
12th grade	27	30	13	28	11	30	
Some college	20	22	11	24	9	24	
**Dental hygiene and history**							
Children receive fluoride varnish							0.01*
Yes	38	49	13	31	18	64	
No	39	51	29	69	10	36	
Household uses fluorinated mouthwash							<0.001*
Yes	39	43	28	61	8	22	
No	51	57	18	39	29	78	
Parents received fluoride education							0.01*
Yes	32	36	18	40	26	72	
No	58	64	27	60	10	28	
Parents report having dental caries							0.21
Yes	77	86	37	80	34	92	
No	13	14	9	20	3	8	
Parents visit the dentist							<0.001*
Yearly	59	66	19	41	33	89	
Never	31	34	27	59	4	11	

*Significant differences were assessed using Fisher’s exact tests*.

*p < 0.05.

Children receiving fluoride varnishing treatments and/or living in households who use mouthwash that contains fluoride were significantly less likely to have dental caries (Table 2), suggesting that the use of fluoride supplements might be protective in reducing dental caries in this sample. Furthermore, children were more likely to have dental caries if their parents reported that they had never visited a dentist. However, children who received fluoride varnishing treatments were more likely to live in a household that uses fluoridated mouthwash (*p* = 0.02), and their parents were more likely to report annual dental visits (*p* = 0.04).

Children were less likely to have cavities if their parents had received education about the importance of fluoride (Table 2), yet, this was not associated with their use of fluoride supplements. Moreover, the majority of parents (78%, 25/32) who reported receiving fluoride education got this information from a dentist or local federally funded program providing free varnishing treatments to children, compared to only 13% (4/32) who received this information from schools and 9% (3/32) from other sources.

There were no associations between reported dental caries in children and the households’ demographic characteristics (Table 2). Similarly, there were no significant associations between report of dental caries in the parents and any of the characteristics (data not shown). However, most of the parents reported having at least one dental caries (86%).

## Discussion

In this study, fluoride detection levels in bottled water were slower than that of tap water, and only 27% of the tap water samples contained fluoride at recommended levels for optimal dental health. With approximately 85% of the population of Nogales, AZ drinking primarily bottled water, it is likely that children and their families in this region are not receiving adequate fluoride for dental health from their drinking water. Furthermore, households were more likely to report that none of the children had experienced dental caries if the children received fluoride varnishing treatments, the household used fluoridated mouthwash, the parents had received education about the importance of fluoride and/or the parents reported yearly dental visits. Overall participants reported that they had not received advice on their drinking water and/or fluoride supplementation from their medical or dental providers. This exploratory study highlights that there is potential for dental health disparities in the U.S.–Mexico border region.

Several studies have shown that lower SES communities, like Nogales, have higher prevalence of dental caries than the general population (2, 28). Counties in Arizona that border Mexico have higher rates of dental caries in children than the state average, and Santa Cruz County, where Nogales is located, has among the highest rates in the state. Because the primary method for ensuring dental health is adequate fluoride intake, in the form of fluoridation of the drinking water, heavy reliance on bottled water was a potential risk for low fluoride exposure (25).

In Nogales, AZ, USA drinking water wells have been closed due to chemical contamination, and there is a constant fear that local municipal tap water may be contaminated. For this reason, many residents choose not to drink or cook with local tap water (25). Bottled water typically contains significantly less fluoride (1, 17), thus increasing dental carie risk. Our analyses describes the potential relationship of fluoride supplementation through varnishing treatments and the household use of fluoridated mouthwash for individuals who primarily consume bottled water or even tap water with inadequate fluoride levels. Until now, most studies have focused on the benefits of fluoridated tap water in reducing dental caries in children (9, 38, 39), but few studies have shown that fluoride supplementation is also associated with lower dental carie rates in individuals who do not have access to or choose not to drink fluoridated tap water (40).

In Nogales, AZ, USA, naturally occurring fluoride in drinking water ranges from 0.22 to 2.90 mg/L and no additional fluoride is added to the tap water. Yet, only 27% of the tap water samples (1 in 6 months) collected had fluoride concentrations above 0.6 mg/L (range undetected to 1.1 mg/L) (Table 1), likely a reflection of the seasonal variation in fluoride concentration (34). Our results mimic those reported on the annual consumer confidence reports for tap water purveyor A (annual average 0.625 mg/L and a range of <0.5–1.0 mg/L), the only water source in which fluoride was detected (41). These findings indicate that children living in the Nogales area may not be receiving recommended levels of fluoride in tap or bottled water for optimal dental health, and that additional interventions for fluoride supplementation are needed.

Our results are consistent with other studies that demonstrate the protective effects of fluoride supplements against dental caries in children (42–44). Of particular importance, is that we demonstrated that fluoride varnishing treatments and the household use of fluoridated mouthwash may significantly reduce dental caries in children who are likely not receiving adequate fluoride in their drinking water. Although, our sample size of 90 might be too small to accurately assess these results, other studies have shown that fluoride supplementation is effective in reducing dental caries, especially in susceptible communities (6, 45, 46).

In addition to these protective measures, yearly dental visits by the parents were also shown to be associated with less dental caries in children. Other studies have shown that access to dental care has not eliminated disparities related to dental caries in children (47, 48). However, our results indicate that those with access to dental care or those who prioritize dental care were more likely to report less dental caries in their children. Interestingly, 18% of our respondents reported being treated for dental caries but never visiting a dentist. One explanation for this can be the use of home remedies, such as cloves (49). In addition, families who purchased mouthwash with fluoride were more likely to report annual dental visits, suggesting that parents may have received this information from their dentists. Furthermore, the children of parents who had received fluoride education were less likely to have caries, suggesting that this information benefited their children. Yet, only 36% reported having received information on the importance of fluoride. Our findings are important and support the need for more detailed information to better understand how individuals in this unique border region are getting treatment for their dental caries, and the type of fluoride education they are receiving and the impact education and fluoride supplement might have on dental health outcomes in the region. Because of the lack of fluoride in both the tap and bottled water in this community, an improved system is needed to reach more families with this information so that this dental health disparity can be addressed in this region and in other communities along both sides of the border.

The exploratory nature of our study has several limitations. First, the study focused solely on bottled water drinkers as an at-risk sample who may benefit from future fluoride interventions, and thus does not reflect the dental health fluoride supplementation of those who primarily drink tap water. Given that the levels of fluoride in tap water were relatively low in this community, it is likely that bottled and tap water drinkers have similar risk for insufficient fluoride intake.

Our study has a small sample size, with only 90 questionnaire responses, although response rates supported validity of results and this is the largest reported on to date in regards to fluoride exposure in the area. Even with this reduced power, several predictors of dental caries in children were identified. We also did not have sufficient power to complete multiple regression analyses that would have allowed us to better assess associations and potential confounding between variables. Future intervention studies should be designed with adequate statistical power to more robustly explore these associations, including evaluation of diet, smoking, and frequency of tooth brushing.

Another limitation is the self-reported nature of much of the data. Although the questionnaire was adapted from validated questionnaires used in previous studies (23, 50), this method of data collection is highly subject to recall bias (51). Further, some of our respondents (*n* = 7) were unable to recall information about their children’s or their own dental histories. In the future, it would be important to obtain information on participants’ dental histories *via* exam or dental records. This would allow for a more robust analysis of this potential health disparity in the border region.

## Conclusion

In conclusion, our exploratory study documented that there is great potential for dental health disparities in children living along the U.S.–Mexico border that should be assessed more thoroughly in future studies. Individuals might be at risk of developing dental caries due to the absence of recommended levels of fluoride in either their bottled or tap water for optimal dental health. None of the bottled water samples and only 27% of the tap water samples were in the range recommended by the ADA. The results from this study also show a lack of fluoride education and use of fluoride supplementation, through varnishing treatments and mouthwash as a preventive measures. In addition, yearly dental visits should be strongly encouraged, and dental and healthcare providers should be provided with guidance to educate individuals about their drinking water and use of fluoride supplements. Future interventions should be developed to promote more federally funded programs that improve dental care access to low-income families and educate them on the importance of fluoride supplementation if they continue to drink and cook with unfluoridated water.

## Ethics Statement

All study procedures were approved by the University of Arizona Human Subject Protection Program. All subjects gave written and informed consent in accordance with the Declaration of Helsinki.

## Author Contributions

PB, KV, NC, and KR contributed to study conception and design and obtained funding; KV, JL, and DL contributed to acquisition of data and analysis; PB, KV, NC, KR, and CT interpreted the data; PB and KV drafted the report; and all the authors contributed to revision of the report and approved the version submitted.

## Conflict of Interest Statement

The authors declare that the research was conducted in the absence of any commercial or financial relationships that could be construed as a potential conflict of interest.
